# The Feasibility of Using Smartphone Sensors to Track Insomnia, Depression, and Anxiety in Adults and Young Adults: Narrative Review

**DOI:** 10.2196/44123

**Published:** 2023-02-17

**Authors:** Doaa Alamoudi, Emma Breeze, Esther Crawley, Ian Nabney

**Affiliations:** 1 Department of Computer Science University of Bristol Bristol United Kingdom; 2 Bristol Medical School University of Bristol Bristol United Kingdom; 3 Centre for Academic Child Health University of Bristol Medical School University of Bristol Bristol United Kingdom

**Keywords:** mHealth, digital, health, mental health, insomnia, technology, sleep, risk, cardiovascular disease, diabetes, men, mortality, sleep disorder, anxiety, depression, heart disease, obesity, dementia, sensor, intervention, young adult

## Abstract

**Background:**

Since the era of smartphones started in early 2007, they have steadily turned into an accepted part of our lives. Poor sleep is a health problem that needs to be closely monitored before it causes severe mental health problems, such as anxiety or depression. Sleep disorders (eg, acute insomnia) can also develop to chronic insomnia if not treated early. More specifically, mental health problems have been recognized to have casual links to anxiety, depression, heart disease, obesity, dementia, diabetes, and cancer. Several researchers have used mobile sensors to monitor sleep and to study changes in individual mood that may cause depression and anxiety.

**Objective:**

Extreme sleepiness and insomnia not only influence physical health, they also have a significant impact on mental health, such as by causing depression, which has a prevalence of 18% to 21% among young adults aged 16 to 24 in the United Kingdom. The main body of this narrative review explores how passive data collection through smartphone sensors can be used in predicting anxiety and depression.

**Methods:**

A narrative review of the English language literature was performed. We investigated the use of smartphone sensors as a method of collecting data from individuals, regardless of whether the data source was active or passive. Articles were found from a search of Google Scholar records (from 2013 to 2020) with keywords including “mobile phone,” “mobile applications,” “health apps,” “insomnia,” “mental health,” “sleep monitoring,” “depression,” “anxiety,” “sleep disorder,” “lack of sleep,” “digital phenotyping,” “mobile sensing,” “smartphone sensors,” and “sleep detector.”

**Results:**

The 12 articles presented in this paper explain the current practices of using smartphone sensors for tracking sleep patterns and detecting changes in mental health, especially depression and anxiety over a period of time. Several researchers have been exploring technological methods to detect sleep using smartphone sensors. Researchers have also investigated changes in smartphone sensors and linked them with mental health and well-being.

**Conclusions:**

The conducted review provides an overview of the possibilities of using smartphone sensors unobtrusively to collect data related to sleeping pattern, depression, and anxiety. This provides a unique research opportunity to use smartphone sensors to detect insomnia and provide early detection or intervention for mental health problems such as depression and anxiety if insomnia is detected.

## Introduction

### Background

Insomnia is defined as inadequate sleep, with the most common causes being poor sleep conditions and stress [1]. It has also been defined as the presence of long sleep latency, also called sleep onset latency, the elapsed time from being fully awake to sleeping [2]. Sleep latency differs from person to person. Sleep latency and how quickly we reach rapid eye movement (REM) sleep can be indicators of the quantity and quality of sleep. Good sleep quality is measured by the time falling asleep (the ideal is 15 to 20 minutes), the ability to stay asleep all night without waking up, and the ability to spend at least 85% of the time asleep rather than awake [3,4].

About 40% of people who are diagnosed with insomnia symptoms also report mental health problems [5]. Mental health problems and insomnia are linked in significant ways, where insomnia is a common diagnostic symptom for depression and anxiety [5]. Compared to the longstanding perspective that regarded sleep issues as related to symptoms of mental problems, there is growing research evidence that the relationship between mental disorders and insomnia is problematic and includes bidirectional causation.

### The Risk of Insomnia

Mendelson et al [6] were the first to publish findings on this topic; they found that over 90% of depressed patients complained about impaired sleep quality. Several early epidemiological studies found similar strong associations: Ford and Kamerow [7], in 1989, found that people with chronic insomnia were 40 times more likely to have major depression and 6 times more likely to have an anxiety disorder. Mellinger et al [8], in 1985, found that there was a significant association between insomnia and depression. This led to the generally accepted concept that insomnia is one of the core symptoms of psychopathology.

**Figure 1:**
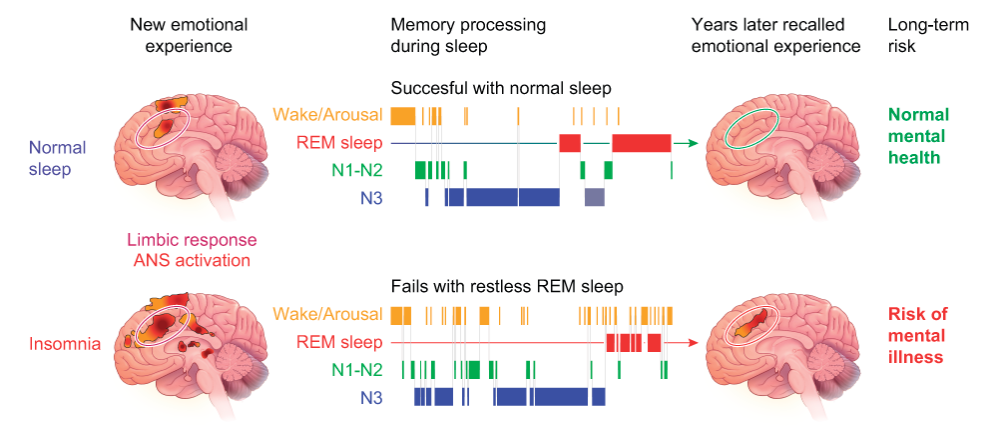
Brain mechanisms of insomnia (adapted from Someren [9], with permission from the American Physiological Society). ANS: autonomic nervous system; REM: rapid eye movement.

Insomnia and depression share multiple underlying mechanisms. Both conditions have been shown to be triggered by psychosocial stressors, which can then cause overactivity of arousal-inducing neurons in the central nervous system (CNS) compared to the sleep-promoting areas, leading to hyperarousal (Figure 1) [9,10]. Another hypothesis is that insomnia could disrupt synaptic plasticity and neural network function, both of which could precipitate depression [11].

More recent studies started to find that insomnia can be an independent indicator for depression. This is highlighted by a 2011 meta-analysis by Baglioni et al [12] of 21 longitudinal epidemiological studies, which found “an overall odds ratio for insomnia to predict depression” of 2.60 (95% CI 1.98-3.42). Results from a 2016 meta-analysis were consistent with these findings (risk ratio 2.27, 95% CI 1.89-2.71) [13]. The recent evidence triggered a change in international guidelines, with insomnia being included in the Diagnostic and Statistical Manual of Mental Disorders, Fifth Edition, a commonly used diagnostic tool, as an independent disorder. This therefore means that insomnia, although still very closely linked to depression, is no longer classified as “primary” or “secondary” and is now considered a disorder in its own right [14,15].

Nyer et al [16] conducted a study to explore the association of sleep disturbance and depressive symptoms in 287 college students with depressive symptoms. The study assumed that students with depressive symptoms and sleep disturbance would have a significant burden of psychiatric symptoms compared to those who had depressive symptoms without sleep disturbance. The study addressed its aims by using different self-report scales, such as the Beck Depression and Anxiety inventory [17,18], the Beck Hopelessness Scale [19], the Anxiety Symptoms Questionnaire [20], the Massachusetts General Hospital Cognitive and Physical Functioning questionnaire [21], and the Quality-Of-Life Enjoyment and Satisfaction questionnaire [22]. For all measures, descriptive statistics showed that the total number of students who had depression with sleep disturbance was 220, and the remaining 67 students had depression without sleep disturbance problems. The results further showed that students who had depressive symptoms with sleep disturbance experienced a significant burden of anxiety symptoms compared to those with depressive symptoms without sleep disturbance [16].

A similar study conducted by Samaranayake et al [23] among university students found that a large number of students were affected by depression and anxiety due to sleep disorders. A total of 1933 students completed a self-report survey. The study found that 39.4% of the students had a sleep disorder lasting over a month. Moreover, depression and anxiety were present in 17.3% and 19.7% of students, respectively, and 7.3% of the students had thought of self-harm.

Studies have shown the close relationship between sleep disorders and mental health problems. Poor sleep quality, defined as waking up frequently during the night, may cause mental health issues such as depression and anxiety. Also, mental health problems could affect an individual’s sleep pattern. Anxiety due to worries or repetitive thoughts might keep their brain awake. These symptoms have to occur at least 3 times a week over a period of at least 3 months in order to be diagnosed as insomnia disorder [11]. Furthermore, studies have shown that insomnia is associated with an increased risk of depression.

## Methods

The main research criterion we looked at in the publications was the use of smartphone sensors to track sleep. We excluded studies that monitored sleep using other tools, such as wearable devices, electroencephalography headsets, or dream headbands. The reason for excluding these factors was the possibility of finding a method that can track sleep unobtrusively. According to Deloitte’s seventh annual mobile consumer survey, around 79% of young adults check their phones before going to sleep [24].

We looked at different studies that monitored sleep using smartphone sensors. The total sample population was up to 205 university students and adults. The designed studies used the Android operating system to collect the data. We categorized the studies based on whether they monitored sleep with a single or multiple sensors.

Anxiety and depression are common, with a prevalence of 18% to 21% among young adults aged 16 to 24 in the United Kingdom [25]. As we wanted to study the relationship between poor sleep and anxiety and depression, we looked to a range of different studies that used mobile sensors to detect individuals’ behavior. They predicted changes in mood using behavioral signals such as location, mobility, speech pattern, phone use, and activities. We excluded papers that discussed other mental health issues, such as bipolar disorder or schizophrenia, and articles that examined changes in mood using other methods, such as physiological and social signals.

## Results

In the field of measuring sleep via smartphone sensors, we found a total of 7 published articles that used mobile sensors to passively collect data [8,14,26-30]. A total of 5 articles discussed the use of mobile sensors to track depression and anxiety [31-35].

### Smartphone Sensors for Sleep Detection

With the widening reach of technology, several groups have carried out sleep studies relying on smartphone sensors [36-38]. The accelerometer embedded in every smartphone has been used to measure phone (hence body) movement to understand sleep stage [27]. Room environment conditions can be measured and monitored by other smartphone sensors to estimate sleep quality. Room environmental variables that can be measured by smartphone sensors to assess the quality of the environment include noise [27,39,40], luminosity or darkness [40], and temperature [41]. Screen on/off timing has been used in several studies as an indicator of sleeping time and duration [27,28,30]. Microphone sensors can also measure an individual’s snoring, which might impact their sleep quality [26]. Snoring can lead to fragmented and unrefreshing sleep, which often causes poor daytime functioning (tiredness and sleepiness) [41]; moreover, snoring is common among people aged 60 years or older [42,43].

### Single-Sensor Method

The iSleep app, which was developed by Hao et al [26], aims to replace wearable devices in sleep tracking by relying on the built-in microphone. The algorithm was able to classify several types of extraneous noise, such as that from fans and air conditioners. However, in order to collect sleep events, the user is required to turn on the app and place the phone in a certain location, and this requires user intervention on a daily basis. That means that in order to calculate sleep duration, the user needs to manually start and close the app. Two matrices (snoring and coughing detection) are used to evaluate the accuracy of the app. During the experiment, the smartphone or tablet is used to collect acoustic data and should be placed 1.5 meters away. A second omnidirectional microphone attached on the headboard and connected to a laptop collects high-quality audio. The microphone should be placed 5 meters away from the bed to avoid noises coming from the laptop fan that might impact the quality of the collected data. Additionally, an iPod is used to record any movement on the bed. The iSleep app uses microphone sensors to collect data related to sleep pattern without considering privacy-sensitive data sets from the user’s smartphone. However, the complicated procedure used to detect sleeping patterns will be difficult to implement in daily life, as it requires a detailed room setup and user intervention, which may be difficult for young adults. Also, although the app has been tested among young adults aged between 20 and 30 years, the prevalence of snoring among young adults is only around 10%, although it increases with age [42,43].

A “tappigraphy” sensor has also been used to measure sleeping patterns [29]. Tappigraphy involves measuring touchscreen events and comparing them with both actigraphy and a daily sleep diary. The study design is based on calculating the number of times the user touches their phone and follows a 24-hour sleep-wake cycle. The longest period of not using the phone is considered to be sleeping time. Tappigraphy overestimates sleeping time compared to actigraphy when a low number of touchscreen touches are measured per day [29]. The sample size of this study was 79 users aged between 16 and 45 years. The large variation in the subjects’ age may have made the data collected an inaccurate reflection of reality, as students and workers have different phone-use styles. Usually, older people spend less time using their phone compared to younger people [44]. The 24-hour sleep-wake cycle might also not have been accurate, especially if the user was a worker or a student, as sometimes, they may need to avoid using their phone during working or class time, affecting the accuracy of predictions of sleeping time, whether this was during the day or the night.

Mobile apps that use screen on/off events to measure sleeping pattern have been discussed in several research papers [28,30,38]. A study based on computing circadian rhythm focused on detecting the chronotype, which means the activity and sleep preferences of an individual within a 24-hour period, and the impact of social jet lag on sleep duration using an unobtrusive and low-cost method based on on/off screen sensors among 9 subjects aged between 19 and 25 years [28]. In order to obtain accurate results, the sleep duration was calculated using a ground truth determined by sleep diaries that were collected daily from participants. When comparing the app performance with the sleep-diary data, there was less than 45 minutes of error. The study showed that participants with an early chronotype experienced the most social jet lag, due to social pressure applied by people with later chronotypes on weekends. However, the study was only interested in determining sleep onset that happened between late night and early morning, without taking into consideration individual preferences. For example, international students who have different sleep times in their home country may adjust their sleep time to be able to contact their family and friends when they are available.

The Know Addiction app [38] was developed to monitor the link between circadian rhythm and individual sleeping patterns using smartphone screen on/off events during a predetermined sleeping window; this window was the period between 10 PM and 10 AM among 61 subjects who were non–shift workers and aged between 20 and 56 years. The app collected total sleep duration according to different parameters. The 3 parameters that were used to measure sleeping pattern were as follows: reactive use episode, proactive use episode, and nonuse episode. A reactive use episode was defined as any notification within 1 minute prior to a screen-on event. In contrast, a proactive use episode was defined as no notifications within 1 minute prior to a screen-on event. The app excluded all reactive use episodes in the sleep indicators calculation. The third parameter was nonuse episodes, defined as a screen on/off event. Sleeping time was determined by measuring the maximal nonuse episode within the predetermined sleeping window. Overall, the study results showed that total daily duration of smartphone use was statistically signiﬁcantly correlated with delayed sleep onset (correlation 0.0808, 95% CI 0.0434-0.1182; *P*<.05) The study was limited to tracking sleeping patterns and did not attempt to measure other health or mental health issues. However, the predetermined sleeping window of 10 PM to 10 AM could not accurately estimate all individuals’ sleep duration, as having mental health problems such as depression or anxiety means that sleep may not occur during the defined sleeping time window. An individual with depression symptoms would prefer to sleep for a longer time than the estimated sleeping time window.

The iSenseSleep app [30] lists all screen on/off events and then analyzes them to estimate sleep duration; it was validated in different groups (4 working mothers and 10 university students). The algorithm provides a list of all time points related to sleep episodes; the iSenseSleep app then considers the longest period to be the sleeping time, ignoring any disturbance that is less than 5 minutes. The app was designed to estimate sleeping time occurring during the night from 10 PM onwards. The app was designed to estimate sleep duration during the normal sleeping time, ignoring those who have changes in their sleeping pattern, for example, weekdays versus weekend days, where the sleeping time may differ. The iSenseSleep app estimates the wake-up time by checking the last screen-on event in the morning that was at least 4 hours since the last screen-off event. The app was able to predict sleep duration with an average error of 24 (SD 17) minutes (7%, SD 4% of the total duration). The estimation of sleep duration was more accurate among university students than working mothers, with an average error of 68 minutes (17% of the total) and 83 minutes (20% of the total), respectively. However, the iSenseSleep app was only used for 2 days to predict the sleeping pattern of the user for the rest of the nights, and that may not reflect the reality of changes in their sleeping pattern over time, especially for people who have problems with depression and anxiety.

Although the above studies conducted their research to understand individual sleeping patterns based on screen on/off events and tappigraphy, sleep quality was not determined in these studies. Sleep quality measures disturbance time, which is when the user wakes up in the middle of the night before going back to sleep. The user can also be asked to enter their sleeping time for working days and weekend days, so their sleeping pattern and sleep quality can be accurately predicted.

### Multiple Sensor Modalities

Chen et al [27] developed Best Effort Sleep (BES), an Android mobile app to estimate sleep duration without user intervention by collecting data from multiple mobile sensors, including the light sensor and the microphone, as well from phone use and whether the device was in stationary mode (ie, not moving). The authors also developed another mobile app, known as Sleep With the Phone (SWP). SWP was developed to collect sleeping data using the accelerometer, with a strict protocol the user needs to follow in order to ensure accuracy while collecting the data, which includes placing the phone on the pillow when the user intends to sleep. Over 1 week with 8 participants, the accuracy of the BES app was tested with SWP and other commercial wearable systems, such as a Jawbone wristband and Zeo headband. When evaluating the accuracy of sleep monitoring using BES, SWP, Jawbone, and Zeo, the authors found that BES achieved a sleep duration error of plus or minus 42 minutes. The use of BES can be considered as an ideal approach to sleep monitoring in terms of its low cost and reduced need for user intervention to record the data, avoiding putting a burden on the user. However, room environment observations might not be considered a good predictor to rely on when predicting sleeping time, as some people may sleep while the room light is turned on. In addition, in the case that the user forgets to recharge the phone, the app will consider that the user is sleeping, as it is in the phone-off mode. The study showed that light and phone-off features contributed to lowering error.

Toss ’N’ Turn (TNT) is an Android mobile app developed to investigate how smartphones can detect bedtime, wake time, and sleep duration without requiring changes in people’s behavior and thus estimate the regularity of sleep quality [36]. A total of 27 participants were studied who were aged between 20 and 59 years. The algorithm observes the sensor logs for the accelerometer, screen on/off events, light, microphone, and battery in a 10-minute window to classify a sleep or not-sleep state. It then eliminates possible sleep detection errors, such as noise or disturbance states, between quiet and stationary situations. The app produces errors of plus or minus 35 minutes, 31 minutes, and 49 minutes in detecting bedtime, wake time, and sleep duration, respectively. However, when the mobile battery is low, the app will stop collecting data, and using multiple sensors can reduce the battery life. The study showed that changes in the screen status (ie, on/off events) and accelerometer were related to wake time, while changes in the light sensor were not always related.

Both studies (ie, BES and TNT) used multiple sensors to understand sleeping patterns, but apps that are based on multiple sensors can reduce the battery life. In addition, both studies showed that the presence of an accelerometer and screen on/off events provided good results in predicting sleeping time, while light sensors could vary in predicting the sleeping pattern. Moreover, the studies used microphone sensors to collect data without leveraging the privacy and sensitivity of the collected data from the users’ smartphones.

Table 1 shows a summary of methods to determine sleep duration; column 3 shows the sample size for validation and column 4 shows the duration of the validation study.

Earlier studies have used unobtrusive methods to predict sleeping patterns without user intervention and have used various sensors to predict sleeping time and various techniques to determine sleep duration and quality. Moreover, earlier papers [28,30,38] have shown that sleep duration, bedtime, and wake time could be identified over a significant period with screen on/off events instead of complex sensors that require additional software, use protocols, or collect sensitive data, such as from the microphone.

**Table 1:** Studies of mobile sensors to monitor sleep.

Study characteristics					Sensors used						
Authors	Year	Sample size, n	Study length, days	Accelerometer		Screen on/off event	Light sensor	Stationary mode	Microphone	Battery	Touch screen event
Hao et al [26]	2013	7	51						✓^a^		
Borger et al [29]	2019	79	1400								✓
Abdullah et al [28]	2014	9	97			✓					
Lin et al [38]	2019	61	14			✓					
Ciman and Wac [30]	2019	14	180			✓					
Chen et al [27]	2016	8	7			✓	✓	✓	✓		
Min et al [36]	2014	27	30	✓		✓	✓		✓	✓	

^a^✓: indicates the type of sensor used in the study.

### Smartphone Sensors for Detecting Depression and Anxiety

The interest in studying the effectiveness of using smartphones for tracking individuals’ sleeping patterns and activities and the relationship with mental well-being has increased at a brisk pace. Over this period of technological advancement, a considerable amount of literature has been published using mobile sensors to categorize mental health and well-being. These studies have used mobile sensors to collect behavioral signals and later relate them to mental health problems.

GPS has been used to study mental health problems such as depression [32,33,35]. These studies discussed a correlation between physical activities and mental health problems. DeMasi et al [32] aimed to track depression symptoms using GPS. Sleep duration was measured by estimating the longest period an individual was not physically active after 9 PM and individual physical activities during the day. A total of 47 undergraduate students installed the app over an 8-week period and completed a self-report survey related to depression and bipolar symptoms. The study showed a positive correlation between physical activities and estimated mental health status. There were limitations to activity recognition, especially that the smartphone was not in a fixed position, participants performed nonstandard activities, and the phones were set down, such as when they were left in a gym locker. Sleep duration and sleep disturbance could not be identified with a single sensor such as GPS.

Saeb et al [33] performed a study of 40 adult subjects over a 2-week period to detect daily life behavior and depression symptoms using GPS, including circadian movement (over 24 hours), location variance, and use of phone features. The data were collected and compared with self-report surveys. The accuracy achieved from this study in predicting depression symptoms through GPS and phone use was 86.5%. A similar study was conducted to study changes in mental health by tracking individuals’ activities and sleeping patterns using mobile sensors [31]. This study aimed to understand changes in depression and stress level using data collected from smartphone sensors. The data were collected from 47 young adults over a 10-week period. GPS was used to track individual daily activities while sleep duration was tracked based on light, microphone data, mobile use, and accelerometer data. The study used the algorithm developed for BES [27] to calculate sleep duration. Self-report surveys of stress and depression were collected on a daily basis. The result of the analysis showed a correlation between individual activities and sleep duration with daily stress and depression level.

StudentLife is an app that is run on smartphones and wearable devices [31,34]. The study was conducted for a 10-week period among a single class of 48 students whose age was between 19 and 30 years. The study aimed to track the engagement and performance of students on an individual level. StudentLife used different types of smartphone sensors, such as the accelerometer, microphone, light sensor, and GPS [31,34]. The microphone was coded to capture sounds every 2 minutes. In contrast, GPS was activated every 10 minutes to calculate the total daily distance moved by the individual. The accelerometer embedded in the smartphone was used to detect the ratio of movement versus being stationary. The study used data retrieved from device lock duration, the accelerometer, the microphone, and the light sensor to calculate sleep duration. A self-report was used to measure mental health status and depression (with the Patient Health Questionnaire-9) [45], perceived stress [46], ﬂourishing [47], and loneliness scales [48]. However, the study did not consider that not carrying the phone during the day would prevent the app from accurately predicting data. For example, if the user left the phone at home, all the data from the microphone and accelerometer would not be collected on that day, and the system would assume that the person was stationary and quiet, which was interpreted as sleeping. In consequence, the predicted mental health state would not be accurate. Other difficulties arose from the app measuring sleep duration using the light level. If the user was from a high-latitude location, which is dark for most of the day, then the app would consider the dark time to be sleeping time. Also, if the user preferred to sleep while keeping the room light on, it would not predict bedtime and sleeping duration accurately.

From the previous studies, we can see that the proliferation of digital technologies and mobile sensors can provide a feasible and unobtrusive method to continuously collect behavioral data from individuals, which can help in better understanding the mental health condition of individuals. Using accelerometers and other phone [31,33] features has been shown to be an efficient way of understanding individuals’ behavior and mental well-being.

## Discussion

### Principal Findings

Mental health problems and sleep are linked in significant ways. Compared to the longstanding perspective that sleep issues are symptoms of mental problems, there is growing research evidence that the relationship between mental disorders and sleep is problematic and includes bidirectional causation.

Many people may not be aware of how their sleeping pattern can impact their health and well-being, seeking treatment only when physical and mental symptoms have started to manifest. Furthermore, both children and adults are reluctant to seek help, with only 1 in 3 receiving treatments for common mental health problems. These reasons, along with the increasing difficulty of accessing primary care services, leave room for an alternative method of insomnia identification. Smartphone sensor technologies in users’ phones may be a suitable method to track sleeping patterns and early sleep disorders. Using these technologies may prevent more serious symptoms arising.

Research has demonstrated the effectiveness of using mobile phone sensors to record personal data and predict mental health and well-being. Several apps have been developed to track behavioral signals and link them with individual mental health and well-being. These apps are based on wearable and nonwearable devices that collect data using accelerometers, microphones, light sensors, and screen on/off events.

### Conclusion

This review describes the effectiveness of several sleep apps that have been used to track insomnia, which can cause depression and anxiety. Furthermore, the studies in this review found that using smartphone sensors to detect mental health problems can be useful for monitoring behavioral patterns that can cause depressive symptoms. Further study is needed in this area to understand the feasibility of using mobile sensors to track sleep disorders and provide early intervention and treatment when insomnia is detected, so as to reduce mental health problems.

## Abbreviations

BES: Best Effort Sleep
CNS: central nervous system
REM: rapid eye movement
SWP: Sleep With the Phone
TNT: Toss ’N’ Turn
